# Venous and Arterial Thrombosis in Ambulatory and Discharged COVID-19 Patients: A Systematic Review and Meta-analysis

**DOI:** 10.1055/a-1913-4377

**Published:** 2022-09-19

**Authors:** Eman M. Mansory, Mohammed Abu-Farhaneh, Alla Iansavitchene, Alejandro Lazo-Langner

**Affiliations:** 1 Department of Medicine, Division of Hematology, Western University, London, Ontario, Canada; 2 Department of Hematology, King Abdulaziz University, Jeddah, Saudi Arabia; 3 Department of Medicine, Western University, London, Ontario, Canada; 4 Health Sciences Library, London Health Sciences Centre, London, Ontario, Canada; 5 Department of Epidemiology and Biostatistics, Western University, London, Ontario, Canada

**Keywords:** thrombosis, ambulatory, outpatients, postdischarge, COVID-19

## Abstract

**Introduction** Venous and arterial thromboses are frequently observed complications in patients with severe novel coronavirus disease 2019 (COVID-19) infection who require hospital admission. In this study, we evaluate the epidemiology of venous and arterial thrombosis events in ambulatory and postdischarge patients with COVID-19 infection.

**Materials and Method** EMBASE and MEDLINE were searched up to July 21, 2021, in addition to other sources. We included studies that assessed the epidemiology of venous and arterial thrombosis events in ambulatory and postdischarge COVID-19 patients.

**Results** A total of 16 studies (102,779 patients) were identified. The overall proportion of venous thromboembolic events in all patients, that is, ambulatory and postdischarge, was 0.80% (95% confidence interval [CI]: 0.44–1.28), 0.28% (95% CI: 0.07–0.64), and 1.16% (95% CI: 0.69–1.74), respectively. Arterial events occurred in 0.75% (95% CI: 0.27–1.47) of all patients, 1.45% (95% CI: 1.10–1.86) of postdischarge patients, and 0.23% (95% CI: 0.019–0.66) of ambulatory patients. The pooled incidence rate estimates per 1,000 patient-days for VTE events were 0.06 (95% CI: 0.03–0.08) and 0.12 (95% CI: 0.07–0.19) for outpatients and postdischarge, respectively, whereas for arterial events were 0.10 (95% CI: 0–0.30) and 0.26 (95% CI: 0.16–0.37).

**Conclusion** This study found a low risk of venous and arterial thrombi in ambulatory and postdischarge COVID-19 patients, with a higher risk in postdischarge patients compared with ambulatory patients. This suggests that regular universal thromboprophylaxis in these patient populations is probably not necessary.

## Introduction

Novel coronavirus disease 2019 (COVID-19) caused by the severe acute respiratory syndrome-coronavirus-2 (SARS-CoV-2) leads to endothelial and coagulation dysfunction which puts patients at an increased risk of both venous and arterial thrombotic events and also increases the morbidity and mortality associated with the disease. 1 Hospitalized COVID-19 patients are at a particularly high risk of thrombosis with estimates of venous thromboembolism (VTE) risk up to 8% in hospitalized patients and 24% in patients admitted to intensive care, according to two large systematic reviews. 2 3 Arterial events are estimated to occur in 1% of ward patients and 5% of critically ill patients. 4 However, the majority of published studies have focused on the incidence of thromboembolism during hospitalization, and it is unclear what the thrombotic risk is in outpatients who have a milder illness and do not require hospitalization and among patients who have been discharged after hospitalization for COVID-19 infection. This information could be helpful in deciding the need for thromboprophylaxis in these patient populations.

## Methods

**Fig. 1:**
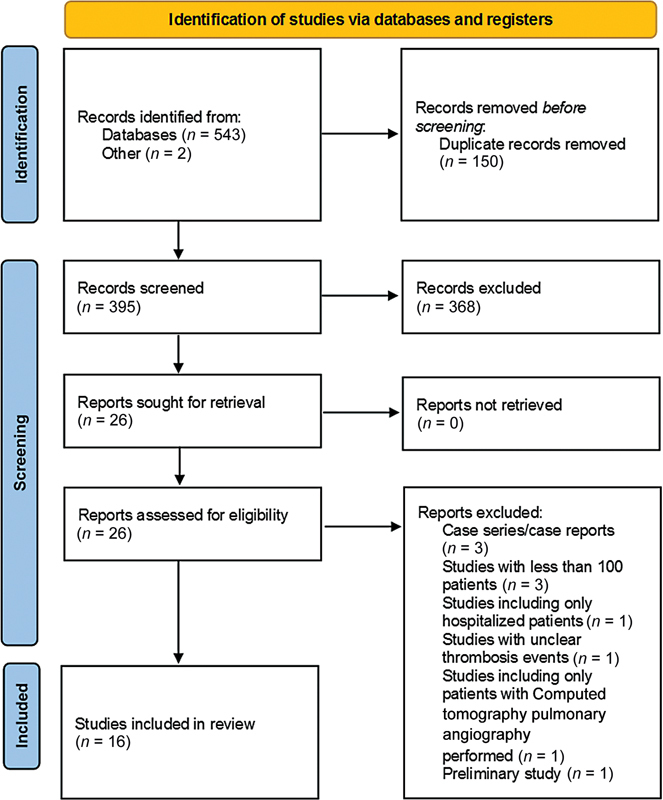
Preferred Reporting Items for Systematic Reviews and Meta-analyses (PRISMA) flow diagram for study selection. CTPA, computed tomography pulmonary angiography.

This review was performed in accordance with the Preferred Reporting Items for Systematic Reviews and Meta-analysis (PRISMA) guidelines ( Fig. 1 ) and is registered in PROSPERO ( *https://www.crd.york.ac.uk/PROSPERO* ; registration number: CRD42021292010).

The literature search was performed using EMBASE and MEDLINE using medical subject headings and free text words through Boolean operators. The retrieved papers were also scanned for additional references in addition to searching preprint databases ( *preprints.org, biorxiv.org* ) for accepted papers not yet published. Full details on the search strategies of the main databases can be found in the Supplementary Material S1 .

We included studies that looked at venous or arterial events in adult patients with COVID-19 either postdischarge, following hospitalization or ambulatory with no history of hospitalization. Full-text articles, abstracts, letters, brief reports, editorials, and correspondence were eligible for inclusion if they reported on randomized control trials (RCTs), observational cohort studies (prospective or retrospective), or case-control studies. We excluded studies with no original data, studies with less than 100 patients and studies that would not reflect the general risk of thrombosis in COVID-19 patients (e.g., studies that only included patients with computed tomography pulmonary angiogram (CTPA)). There was no language restriction.

We conducted an initial broad screening according to title and reviewed all abstracts judged to be relevant. Potentially relevant papers were reviewed in total length. Articles were independently assessed by two reviewers (E.M.M. and M.A.-F) to verify eligibility and decide on inclusion vs exclusion. Disagreements were resolved by consensus or in conjunction with a third reviewer (A. L.-L.). Translation of included papers from Dutch to English was conducted using Google Chrome's built-in translation tool.

We extracted data on study characteristics, study methodology (including sample size, study design, health care setting, and ultrasound screening strategy), patients and diseases (hospitalization, thrombosis risk factors, and thromboprophylaxis strategy), and outcomes including (venous thromboembolism or arterial thrombosis.

The study aimed to look at the proportion of venous thromboembolism (i.e., deep venous thrombosis and pulmonary embolism) or arterial thrombosis in outpatients or postdischarge patients with COVID-19 infection as the primary outcome. We used the proportion of patients diagnosed with a thrombotic event in the included studies (prevalence) to compensate for the differences in event reporting between studies. Some studies used prevalence, while others used incidence rates depending on the study type. If needed, authors were contacted to obtain missing data. Additionally, we also estimated incidence rates if possible.

The quality and risk of bias of included observational studies were assessed using the Newcastle–Ottawa Scale (NOS) to evaluate the quality of nonrandomized studies in meta-analyses. 5 For randomized trials, we planned to use the Jadad scale. 6

### Statistical Analysis

A meta-analysis of proportions was done for the frequency of VTE and arterial thrombosis. We estimated pooled proportions through a Freeman–Tukey transformation, using fixed and random effects models. We also estimated incidence rates (IR) from imperfect data assuming no losses to follow-up for the reported observation periods. We applied standard methods to obtain 95% confidence intervals (CIs) for the IR that we used to estimate standard errors. We calculated pooled estimates for the IR using the generic inverse variance method. 7

The reported pooled proportions were obtained by a random-effects model given statistical heterogeneity. Sensitivity analyses were performed according to setting (outpatients vs. postdischarge), thrombosis type (VTE vs. arterial), and study type (retrospective vs. prospective). Additionally, we conducted a meta-analysis of the relative risk for VTE for the studies that included both postdischarge and outpatients. Exploratory analyses were also conducted according to percentage of use of thromboprophylaxis (<20 vs. >20%) and duration of follow-up in the study (<42 vs. > 42 days). Heterogeneity between studies was evaluated using Cochrane Q and Higgins *I* ^2^ tests. Publication bias was assessed using Egger's tests and funnel plots. The analysis was done using Microsoft Excel 365 (Microsoft Corp. Redmond, Washington, United States) and MedCalc Statistical Software version 19.2.6 (MedCalc Software Ltd., Ostend, Belgium).

## Results

### Search Strategy and Included Studies

The database search included papers published up to July 21, 2021. The search produced 543 studies through Medline and Embase and an additional two articles from other sources. A total of 368 studies were excluded after title and abstract screening, and a total of 26 articles were reviewed in full text. Of those, 16 studies met our eligibility criteria and were included. The reasons for excluding studies are summarized in Fig. 1 . All the included studies were cohort studies (11 retrospective and 5 prospective). All studies were in English except one study published in Dutch. 8

The studies were conducted in North America (seven in the United States and one in Canada), Europe (three from the United Kingdom and one each from Norway, the Netherlands, Denmark, and Belgium), and there was one multicenter study. Ten studies reported events in postdischarge patients, three reported on outpatients, and three reported on both outpatients, and postdischarge. The follow-up duration in the studies ranged from 30 days up to 6 months.

Only one study performed regular VTE screening at 6-week postdischarge. 9 With regard to thromboprophylaxis, many studies used some form of thromboprophylaxis on an individual basis or according to risk scores. 10

Characteristics of included studies are shown in Table 1 . Details of the included papers and a detailed reference list can be found in the Supplementary Tables S1 and S2 .

**Table 1:** Characteristics of included studies

Characteristics	No of studies
Country	
North America	8
Europe	7
Multicenter study	1
Study design	
Prospective cohort	5
Retrospective cohort	11
Setting	
Outpatients only	3
Postdischarge only	10
Both	3
Events reported	
VTE events only	8
Both Venous and arterial	8
Thromboprophylaxis strategy	
Prophylaxis used	7
No prophylaxis used	9

Abbreviation: VTE, venous thromboembolism.

All estimates showed high statistical heterogeneity; therefore, we focused our analysis on the results of random effects estimates, although the fixed effects estimates are shown for completeness when appropriate. Funnel plots are included in the Supplementary Figs. S1 to S8 .

### Venous Thromboembolic Events

The results are summarized in Table 2 . The 16 included studies reported on 102,779 patients diagnosed with COVID-19 infection treated as either outpatients (85,051) or posthospital discharge (17,728). In total, 388 patients had a VTE event with a pooled prevalence estimate of 0.80% (95% CI:0.44–1.28; Q-test: *p* < 0.0001, *I* ^2^ = 97.33%).

**Table 2:** Estimates of the proportion of venous and arterial thrombotic events in ambulatory and postdischarge patients with COVID-19 infection

Patient population	No. of total patients	Percentage of patients with VTE (%)	95% confidence interval
Venous thromboembolic events			
All patients	102,779	0.80	0.44–1.28
Postdischarge	17,728	1.16	0.69–1.74
Outpatients	85,051	0.28	0.07–0.64
Subanalysis according to study design			
Prospective studies	14,904	1.37	0.48–2.69
Retrospective studies	87,875	0.62	0.25–1.14
Arterial events			
All patients	20,187	0.75	0.27–1.47
Postdischarge	7,816	1.46	1.10–1.86
Outpatients	12,371	0.23	0.02–0.66
Subanalysis according to study design			
Prospective studies	13,889	0.71	0.01–3.09
Retrospective studies	6,298	0.78	0.34–1.39

Abbreviations: COVID-19, novel coronavirus disease 2019; VTE, venous thromboembolism.

In the outpatient population, 141 patients developed a VTE event with a pooled prevalence estimate of 0.28% (95% CI: 0.07–0.64, *p* < 0.0001, *I* ^2^ = 97.45%). Among the postdischarge patients, 247 developed a VTE event with a pooled prevalence estimate of 1.16% (95% CI: 0.69–1.73; Q-test: *p* < 0.001, *I* ^2^ = 87.21%).

Prospective studies included 14,904 patients with a pooled VTE prevalence estimate of 1.37% (95% CI: 0.48–2.69; Q-test: *p* < 0.001, *I* ^2^ = 94.75%) and retrospective studies included 87,875 patients with a pooled VTE prevalence estimate of 0.62% (95% CI: 0.25–1.14; Q-test: *p* < 0.001, *I* ^2^ = 97.63%).

Estimates for the pooled IR of VTE and arterial events are shown in Table 3 . Funnel and forest plots are included in the Supplementary Figs. S9 to S16 . The estimated pooled IR for VTE events in outpatients was 0.06 (95%CI: 0.03–0.08) events per 1,000 patient-days of observation and the estimate for postdischarge patients was 0.12 (0.07–0.19).

**Table 3:** Pooled estimates for incidence rate of venous and arterial thrombotic events in ambulatory and postdischarge patients with COVID-19 infection

	IR (events per 1000 patient-days)		Heterogeneity	
	IR	95% CI	Q-test ( *p* )	Higgins *I* ^2^ (%)
Venous thromboembolic events (outpatients)				
Fixed effects	0.02	0.02–0.03	<0.001	95.5
Random effects	0.06	0.03–0.08		
Venous thromboembolic events (postdischarge)				
Fixed effects	0.06	0.04–0.07	<0.001	82.7
Random effects	0.12	0.07–0.19		
Arterial events (outpatients)				
Fixed effects	0.01	0–0.01	0.001	93.2
Random effects	0.10	0–0.30		
Arterial events (postdischarge)				
Fixed effects	0.21	0.17–0.25	0.182	35.8
Random effects	0.26	0.16–0.37		

Abbreviations: CI, confidence interval; COVID-19, novel coronavirus disease 2019; IR, incidence rates.

**Fig. 2:**
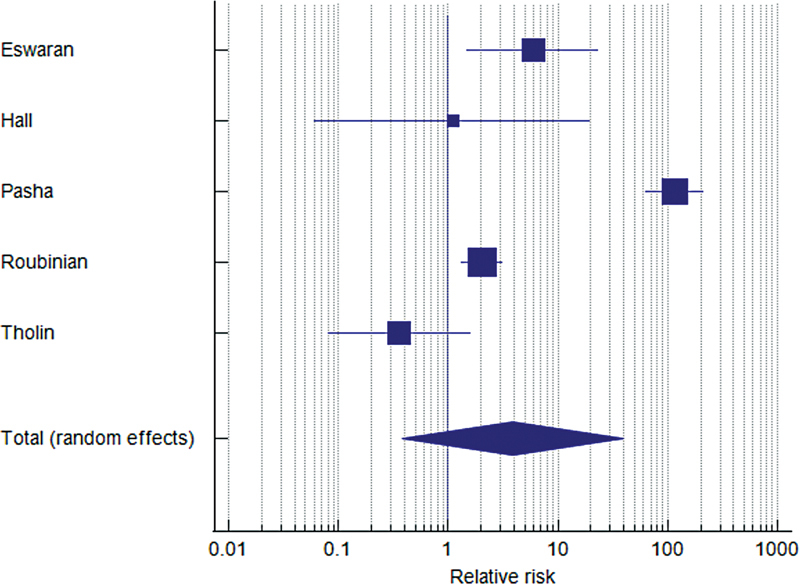
Forest plot for the risk of venous thromboembolism in discharged patients compared with outpatients. Forest plot showing the pooled relative risk estimate of VTE for COVID-19 patients discharged from hospital compared with outpatients. The pooled relative risk was 3.87 (95% CI: 0.38–39.18; *p* = 0.252) with a high heterogeneity (Cochrane's Q = 128.34 [ *p* < 0.001]; Higgins' *I* ^2^ = 96.88 (95% CI: 94.82–98.13). CI, confidence interval; COVID-19, novel coronavirus disease 2019; VTE, venous thromboembolism.

A meta-analysis conducted for studies including both discharged patients and outpatients showed that, compared with the latter, the pooled relative risk of VTE among discharged patients was 3.87 (95% CI: 0.38–39.18; *p* = 0.252) with a high heterogeneity (Cochrane's *Q* = 128.34 ( *p* < 0.001); Higgins' *I* ^2^ = 96.88 (95% CI: 94.82–98.13; Fig. 2 ). Additional comparisons according to study design showed that in prospective studies the relative risk for discharged patients compared with outpatients was 0.46 (95% CI: 0.12–1.72; *p* = 0.246; Q-test: *p* = 0.498; *I* ^2^ = 0%; *n* = 890). In comparison, the relative risk reported in retrospective studies was 11.31 (95% CI: 0.57–224.85; *p* = 0.112; Q-test: *p* < 0.001, *I* ^2^ = 98.23; *n* = 82,666). Estimates of VTE events according to the reported percent of utilization of thromboprophylaxis in studies, as well as duration of follow-up showed similar results among groups ( Supplementary Table S3 ).

### Arterial Events

Studies that evaluated arterial events included a total of 20,187 patients (including both outpatients and postdischarge) with an overall proportion of arterial events of 0.75 (95% CI: 0.27–1.47; Q-test: *p* < 0.001, *I* ^2^ = 94.52%). In the outpatient population (12,371 patients) arterial thromboses occurred in 0.23% (95% CI: 0.019–0.66; Q-test: *p* < 0.001, *I* ^2^ = 88.85%). Among 7,816 postdischarge patients, arterial events occurred in 1.46% (95% CI: 1.10–1.86; Q-test: *p* = 0.242, *I* ^2^ = 25.57%).

Retrospective studies included 6,298 patients with a pooled proportion of arterial events of 0.77% (95% CI: 0.34–1.39; Q-test: *p* = 0.001, *I* ^2^ = 77.81%), while prospective studies included 13,889 patients with a pooled proportion of arterial events of 0.71% (95% CI: 0.01–3.09; Q-test: *p* < 0.0001, *I* ^2^ = 99.11%).

Estimates for the pooled IR of arterial events per 1,000 patient-days of observation were 0.10 (95%CI: 0–0.303) in outpatients and 0.26 (95% CI: 0.16–0.37) in postdischarge patients, respectively. Given a lack of data, we were unable to conduct a meta-analysis comparing discharged patients to outpatients.

## Discussion

Hospitalization for COVID-19 infection is associated with an increased risk of thrombosis, 2 3 but whether the COVID-19 infection increases the risk of VTE outside of the hospital setting is not well established. In this systematic review, we found that patients with COVID-19 infection not severe enough to require hospitalization or those, who are discharged and not in the acute phase anymore, do not have a markedly increased risk of thrombosis, both venous and arterial and in the case of postdischarge patients, their risk might be similar to other medically ill outpatients after hospital discharge. The MARINER trial looked at the benefit of using Rivaroxaban 10 mg. daily for thromboprophylaxis after hospital discharge in medical patients at high risk of VTE based on a score of ≥4 in the modified IMPROVE-VTE score or ≥2 plus D-Dimer level > 2 ULN (upper limit of normal) at discharge. In this study, the occurrence of symptomatic VTE in the placebo and intervention groups were 1.10 and 0.83%, respectively. 11 For comparison, the occurrence of VTE events is more than double (up to 2.8%) in patients undergoing major orthopedic surgery which is an accepted indication for anticoagulant prophylaxis. 12 More recently, the benefit of anticoagulant prophylaxis to reduce mortality in populations at risk for VTE has been questioned as the use of anticoagulants is associated to a higher risk of bleeding counterbalancing the relevance of VTE prevention. 13 Furthermore, our results showed that compared with fully ambulatory patients, the relative risk for VTE among discharged patients was not statistically significant, despite being numerically higher. Additionally, exploratory analyses showed similar proportion of VTE in patients according to the reported proportion of use of thromboprophylaxis in individual studies. These and the previously mentioned findings are important, since the use of anticoagulant prophylaxis in all these patients would be of doubtful benefit with the reported rates of events and uncertainty about the increased risk of VTE, and it would likely result in a higher risk of bleeding complications.

The findings of our review are consistent with other reports and expand on the information reported by another systematic review that also looked at the risk of VTE in postdischarge COVID-19 patients and reported a VTE risk of 1.8% (95% CI: 0.8–4.1%, *I* ^2^ = 96.0%), 14 in agreement with our findings. We believe that the difference in calculated risk stems from including two recent large cohorts in our systematic review that were not included in the previous one. 15 16 Additionally, our study reports on pooled estimates of incidence rates which also demonstrate a low IR of venous and arterial events.

Although some studies comparing COVID-19-positive with COVID-19-negative patients have reported a higher risk of thrombosis in COVID-19 patients compared with other medical patients, the results were not statistically significant. One study assessed the occurrence of postdischarge VTE in patients with COVID-19 compared with a 2019 control cohort and reported an odds ratio (OR) of 1.6 (95% CI: 0.8–3.1). 17 Another study that looked at the incidence of complications in the post–acute phase of SARS-CoV-2 infection in outpatients, comparing SARS-CoV-2-positive with SARS-CoV-2-negative individuals, found the VTE risk to be slightly higher in COVID-19 infection with 0.2 versus 0.1% (risk difference = +0.1% [95% CI: 0.0–0.2]; risk ratio = 1.77 [95% CI: 1.09–2.86]). 18 Pasha et al showed that the incidence of VTE increased within the first week following positive COVID-19 testing and the rate then decreased and returned to baseline by the sixth week; however, in this study, only 3% of VTE events occurred in patients who were never hospitalized. 15 In ambulatory patients, a randomized trial including 657 symptomatic outpatients with COVID-19 evaluated the use of different thromboprophylaxis strategies compared with placebo. 19 The study was terminated early because of a very low event rate. Among randomized participants who were on aspirin (81-mg once daily), apixaban (2.5-mg twice daily), apixaban (5.0-mg twice daily), or placebo, the rates of composite outcomes (including symptomatic venous or arterial thromboembolism, or hospitalization for cardiac or pulmonary cause) after 45 days were 0.0, 0.7, 1.4, and 0.0%, respectively, with no significant differences between the active groups and the placebo group. Interestingly, a study on outpatients with COVID-19 who were on outpatient anticoagulation at the time of diagnosis reported a reduction in the risk of hospitalization but not mortality. 20

It is estimated that arterial events complicate 3.9% of COVID-19 admissions, although the amount of literature concerning arterial events is more limited. 21 Similarly, the burden in non-COVID-19 patients is unknown. A study investigating the rates of myocardial infarctions and stroke following hospital admission for pneumonia found an incidence of 2.5% for myocardial infarction and 0.2% for stroke. 22 The estimate for the risk of cardiovascular events in general medically ill outpatients was unclear.

It has been suggested that the administration of low molecular weight heparin during the earlier phases of COVID-19 infection may be beneficial not only in the prevention of thrombosis risk but also in reducing systematic and pulmonary inflammation and limiting viral invasion. 23 24 A randomized controlled trial, the OVID trial (ClinicalTrials.gov Identifier: NCT04400799), is currently being conducted. This study aims to evaluate whether prophylactic-dose enoxaparin (vs. no treatment) can decrease early all-cause mortality and subsequent hospitalizations in adult symptomatic ambulatory COVID-19 patients with no other indications to receive anticoagulation. 25

The use of thromboprophylaxis in postdischarge patients has been explored previously in patients with medical illness by the MARINER trial which showed no reduction in the risk of symptomatic venous thromboembolism and death due to venous thromboembolism compared with placebo. 11 A recent randomized control study applied the IMPROVE-VTE score on discharged patients posthospitalization with COVID-19 and reported that high-risk patients with a score of ≥4, or 2 to 3 with a D-dimer >500 ng/mL, had improved outcomes when on thromboprophylaxis with rivaroxaban 10 mg/day for 35 days. 26 This study used a composite outcome including symptomatic, asymptomatic, and fatal VTE (all detected by mandatory screening), as well as arterial thromboembolism including myocardial infarction, stroke, adverse limb events, and cardiovascular death. The generalizability of these findings is questionable for several reasons. The study accrued only 30% of potentially eligible patients, and more than half of the patients in this study had been admitted to the intensive care unit suggesting that the population included a larger than usual proportion of patients with severe disease. Additionally, the reported proportion of events in this study amounted to near 10% in the control arm which is not in agreement with large observational studies suggesting that the included population in the trial was a higher risk one. However, the findings of the trial raise the question about the need to identify specific subpopulations of patients at a higher risk of postdischarge VTE and that might benefit from thromboprophylaxis.

Current guidelines suggest against (1) using thromboprophylaxis in medical outpatients with no major provoking risk factors for VTE, and (2) using extended-duration outpatient VTE prophylaxis in the case of discharged patients. 27 Most importantly, the American College of Chest Physicians (ACCP) and the American Society of Hematology recommendations suggest that routine extended thromboprophylaxis after hospital discharge of COVID-19 patients may not have a net clinical benefit. 28 29

Our findings support these recommendations as the proportion and incidence of venous and arterial thrombosis observed in our study are similar to the reported risk of bleeding associated with the use of thromboprophylaxis, in addition to the added burden and cost. This aligns with the findings of a study by Giannis et al who reported that patients discharged on anticoagulation had a major bleeding rate of 2.45% compared with 1.63% in those who were discharged without any anticoagulation. 10 Therefore, the decision to use thromboprophylaxis should not be influenced by COVID-19 status but should be based on an overall assessment of patients' thrombosis risk factors balanced with their bleeding risk, although a precise and proven algorithm to assess these risks is currently not available.

## Limitations

A limitation to this meta-analysis is that the included studies for postdischarge patients had different rates of patients on prophylactic anticoagulation ranging from 0 to 97%, but in general, there was only a small percentage of high-risk discharged patients started on prophylactic anticoagulation, but no clear criteria were used. Given the heterogeneity in approaches, subgroup analyses might be misleading and and need to be interpreted with much caution. Additional limitations to our study are those inherent to the included reports, mainly the fact that the majority of patients were included in retrospective studies. However, the proportion of events was almost double in prospective studies compared with retrospective ones, suggesting the possibility of recall bias, although the overall incidence is still very low even considering the possibility of events underreporting. Additionally, the estimate of IR needs to be interpreted with caution, as it was conducted under the assumption of no losses to follow-up which is unlikely to be the case. However, given the short observation period (less than 42 days in 11 of 16 studies) losses to follow-up are likely low and would result in marginal modifications to the estimates. In any case, the proportions of events were similar in studies with shorter or longer follow-up. Another issue is the potential for COVID-19 vaccines to influence the risk of VTE. In this regard, most recent studies have reported no influence of vaccines on the incidence of VTE, 30 31 except perhaps for the ChAdOx1 vaccine, 32 but overall it seems that vaccines are safe in this regard. This information was not available in the studies included in this review. Finally, although statistical heterogenicity was found between studies, all the proportion estimates were very low, and there was no evidence of publication bias for most outcomes ( Supplementary Table S4 ).

## Conclusion

In conclusion, our findings suggest that patients with COVID-19 infection are not severe enough to require hospitalization and those who are postdischarge and past the acute phase are not at a particular high rate of thrombosis, and their thrombosis risk is similar to other patients with medical illness. We believe that these findings support the current recommendations, suggesting that prophylactic anticoagulation should not be routinely prescribed in these groups of patients. However, to better inform clinical decisions, further research is needed to define subgroups that could potentially benefit from anticoagulant prophylaxis by identifying those patients at a high risk of postdischarge thrombosis and a low bleeding risk that would derive the most benefit from this intervention.

## References

[1] Salabei J K Fishman T J Asnake Z T Ali A Iyer U G COVID-19 Coagulopathy: Current knowledge and guidelines on anticoagulation Heart Lung 2021 50 02 357 360 33524866 10.1016/j.hrtlng.2021.01.011 PMC7816593

[2] Mansory E M Srigunapalan S Lazo-Langner A Venous thromboembolism in hospitalized critical and noncritical COVID-19 patients: a systematic review and meta-analysis TH Open 2021 5 03 e286 e294 34240001 10.1055/s-0041-1730967 PMC8260281

[3] Nopp S Moik F Jilma B Pabinger I Ay C Risk of venous thromboembolism in patients with COVID-19: A systematic review and meta-analysis Res Pract Thromb Haemost 2020 4 07 1178 1191 33043231 10.1002/rth2.12439 PMC7537137

[4] Malas M B Naazie I N Elsayed N Mathlouthi A Marmor R Clary B Thromboembolism risk of COVID-19 is high and associated with a higher risk of mortality: A systematic review and meta-analysis EClinicalMedicine 2020 29 100639 33251499 10.1016/j.eclinm.2020.100639 PMC7679115

[5] Wells G A Shea B O'Connell D The Newcastle-Ottawa Scale (NOS) for assessing the quality of nonrandomised studies in meta-analysesAccessed August 16, 2022 at:https://www.ohri.ca/programs/clinical_epidemiology/oxford.asp

[6] Jadad A R Moore R A Carroll D Assessing the quality of reports of randomized clinical trials: is blinding necessary? Control Clin Trials 1996 17 01 1 12 8721797 10.1016/0197-2456(95)00134-4

[7] Lane P W Meta-analysis of incidence of rare events Stat Methods Med Res 2013 22 02 117 132 22218366 10.1177/0962280211432218

[8] Leijte W T Wagemaker N MM van Kraaij T DA Mortality and re-admission after hospitalization with COVID-19 [in Dutch] Ned Tijdschr Geneeskd 2020 164 49 D5423 33332036

[9] Engelen M M Vandenbriele C Balthazar T Venous thromboembolism in patients discharged after COVID-19 hospitalization Semin Thromb Hemost 2021 47 04 362 371 33893631 10.1055/s-0041-1727284

[10] Giannis D Allen S L Tsang J Postdischarge thromboembolic outcomes and mortality of hospitalized patients with COVID-19: the CORE-19 registry Blood 2021 137 20 2838 2847 33824972 10.1182/blood.2020010529 PMC8032474

[11] MARINER Investigators Spyropoulos A C Ageno W Albers G W Rivaroxaban for thromboprophylaxis after hospitalization for medical illness N Engl J Med 2018 379 12 1118 1127 30145946 10.1056/NEJMoa1805090

[12] White R H Romano P S Zhou H Rodrigo J Bargar W Incidence and time course of thromboembolic outcomes following total hip or knee arthroplasty Arch Intern Med 1998 158 14 1525 1531 9679793 10.1001/archinte.158.14.1525

[13] Klemen N D Feingold P L Hashimoto B Mortality risk associated with venous thromboembolism: a systematic review and Bayesian meta-analysis Lancet Haematol 2020 7 08 e583 e593 32735837 10.1016/S2352-3026(20)30211-8

[14] Zuin M Engelen M M Barco S Incidence of venous thromboembolic events in COVID-19 patients after hospital discharge: a systematic review and meta-analysis Thromb Res 2022 209 94 98 34896917 10.1016/j.thromres.2021.11.029 PMC8648604

[15] Pasha A K McBane R D Chaudhary R Timing of venous thromboembolism diagnosis in hospitalized and non-hospitalized patients with COVID-19 Thromb Res 2021 207 150 157 34649175 10.1016/j.thromres.2021.09.021 PMC8495042

[16] Roubinian N H Dusendang J R Mark D G Incidence of 30-Day venous thromboembolism in adults tested for SARS-CoV-2 infection in an integrated health care system in Northern California JAMA Intern Med 2021 181 07 997 1000 33818615 10.1001/jamainternmed.2021.0488 PMC8022258

[17] Roberts L N Whyte M B Georgiou L Postdischarge venous thromboembolism following hospital admission with COVID-19 Blood 2020 136 11 1347 1350 32746455 10.1182/blood.2020008086 PMC7483432

[18] Lund L C Hallas J Nielsen H Post-acute effects of SARS-CoV-2 infection in individuals not requiring hospital admission: a Danish population-based cohort study Lancet Infect Dis 2021 21 10 1373 1382 33984263 10.1016/S1473-3099(21)00211-5 PMC8110209

[19] ACTIV-4B Investigators Connors J M Brooks M M Sciurba F C Effect of antithrombotic therapy on clinical outcomes in outpatients with clinically stable symptomatic COVID-19: the ACTIV-4B randomized clinical trial JAMA 2021 326 17 1703 1712 34633405 10.1001/jama.2021.17272 PMC8506296

[20] Hozayen S M Zychowski D Benson S Outpatient and inpatient anticoagulation therapy and the risk for hospital admission and death among COVID-19 patients EClinicalMedicine 2021 41 101139 34585129 10.1016/j.eclinm.2021.101139 PMC8461367

[21] Tan B K Mainbourg S Friggeri A Arterial and venous thromboembolism in COVID-19: a study-level meta-analysis Thorax 2021 76 10 970 979 33622981 10.1136/thoraxjnl-2020-215383

[22] Perry T W Pugh M JV Waterer G W Incidence of cardiovascular events after hospital admission for pneumonia Am J Med 2011 124 03 244 251 21396508 10.1016/j.amjmed.2010.11.014 PMC3061467

[23] Lang J Yang N Deng J Inhibition of SARS pseudovirus cell entry by lactoferrin binding to heparan sulfate proteoglycans PLoS One 2011 6 08 e23710 21887302 10.1371/journal.pone.0023710 PMC3161750

[24] Tang N Bai H Chen X Gong J Li D Sun Z Anticoagulant treatment is associated with decreased mortality in severe coronavirus disease 2019 patients with coagulopathy J Thromb Haemost 2020 18 05 1094 1099 32220112 10.1111/jth.14817 PMC9906401

[25] Barco S Bingisser R Colucci G Enoxaparin for primary thromboprophylaxis in ambulatory patients with coronavirus disease-2019 (the OVID study): a structured summary of a study protocol for a randomized controlled trial Trials 2020 21 01 770 32907635 10.1186/s13063-020-04678-4 PMC7479300

[26] MICHELLE investigators Ramacciotti E Barile Agati L Calderaro D Rivaroxaban versus no anticoagulation for post-discharge thromboprophylaxis after hospitalisation for COVID-19 (MICHELLE): an open-label, multicentre, randomised, controlled trial Lancet 2022 399(10319):50 59 34921756 10.1016/S0140-6736(21)02392-8 PMC8673881

[27] Schünemann H J Cushman M Burnett A E American Society of Hematology 2018 guidelines for management of venous thromboembolism: prophylaxis for hospitalized and nonhospitalized medical patients Blood Adv 2018 2 22 3198 3225 30482763 10.1182/bloodadvances.2018022954 PMC6258910

[28] Cuker A Tseng E K Nieuwlaat R American Society of Hematology living guidelines on the use of anticoagulation for thromboprophylaxis in patients with COVID-19: July 2021 update on postdischarge thromboprophylaxis Blood Adv 2022 6 02 664 671 34727173 10.1182/bloodadvances.2021005945 PMC8566097

[29] Moores L K Tritschler T Brosnahan S Prevention, diagnosis, and treatment of VTE in patients with coronavirus disease 2019: CHEST Guideline and Expert Panel Report Chest 2020 158 03 1143 1163 32502594 10.1016/j.chest.2020.05.559 PMC7265858

[30] Tanislav C Rosenbauer J Zingel R Kostev K No increased incidence of venous thrombosis or pulmonary embolism after SARS-CoV-2 vaccination in Germany Public Health 2022 207 14 18 35461122 10.1016/j.puhe.2022.03.004 PMC8923878

[31] Houghton D E Wysokinski W Casanegra A I Risk of venous thromboembolism after COVID-19 vaccination J Thromb Haemost 2022 20 07 1638 1644 35398975 10.1111/jth.15725 PMC9115120

[32] Andrews N J Stowe J Ramsay M E Miller E Risk of venous thrombotic events and thrombocytopenia in sequential time periods after ChAdOx1 and BNT162b2 COVID-19 vaccines: A national cohort study in England Lancet Reg Health Eur 2022 13 100260 34927118 10.1016/j.lanepe.2021.100260 PMC8668159

