# Supplementary material for: Venous and Arterial Thrombosis in Ambulatory and Discharged COVID-19 Patients: A Systematic Review and Meta-analysis
Source: TH Open. 2022 Sep 19;6(3):e276–82. doi: 10.1055/a-1913-4377 (PMC9484870; doi:10.1055/a-1913-4377)
Supplement: Supplementary file 1 — Supplementary Material [file 10-1055-a-1913-4377-s220012.pdf]

Supplementary Material S1

Search Strategy

Database: Ovid MEDLINE(R) ALL <1946 to July 21, 2021>

Search Strategy:

1. ((((exp Coronavirus/or exp Coronavirus Infections/or (D614G or coronavirus* or corona virus* or OC43 or NL63 or 229E or HKU1 or HCoV* or ncov* or covid* or sars-cov* or sarscov* or Sars-coronavirus* or Severe Acute Respiratory Syndrome Coronavirus*).mp.) and ((20191* or 202*).dp. or 20190101:20301231.(ep).)) not (SARS or SARS-CoV or MERS or MERS-CoV or Middle East respiratory syndrome or camel* or dromedar* or equine or coronary or coronal or covidence* or covidien or influenza virus or HIV or bovine or calves or TGEV or feline or porcine or BCoV or PED or PEDV or PDCoV or FIPV or FCoV or SADS-CoV or canine or CCov or zoonotic or avian influenza or H1N1 or H5N1 or H5N6 or IBV or murine corona*).mp.) or ((((pneumonia or covid* or coronavirus* or corona virus* or ncov* or 2019-ncov or sars*).mp. or exp pneumonia/) and Wuhan.mp.) or (2019-ncov or ncov19 or ncov-19 or 2019-novel CoV or sars-cov2 or sars-cov-2 or sarscov2 or sarscov-2 or Sars-coronavirus2 or Sars-coronavirus-2 or SARS-like coronavirus* or coronavirus-19 or covid19 or covid-19 or covid 2019 or ((novel or new or nouveau) adj2 (CoV on nCoV or covid or coronavirus* or corona virus or Pandemi*2)) or ((covid or covid19 or covid-19) and pandemic*2) or (coronavirus* and pneumonia)).mp. or COVID-19.rx,px,ox. or severe acute respiratory syndrome coronavirus 2.os.)) and 20191201:20301231.(dt). (160097)
2. Venous Thromboembolism/or exp venous thrombosis/or exp Pulmonary Embolism/ (97997)
3. (thrombos$ adj3 (venous or vein$1 or deep-vein$ or deep-venous)).tw,kf. (58712)
4. (thrombo?emboli$ adj3 (venous or vein$1)).tw,kf. (26908)
5. ((pulmona$ or lung$) adj (embol$ or thrombo?embol$ or micro?embol$)).tw,kf. (46392)
6. (VTE or DVT or (thromb$ adj3 (generati$ or risk$1))).tw,kf. (50967)
7. or/2-6 (166923)
8. exp Ambulatory Care/or ambulatory care facilities/or exp community health centers/or exp outpatient clinics, hospital/or surgicenters/or outpatients/(117754)
9. (outpatient$ or out-patient$ or ((follow-up or followed or followup) adj3 clinic$1) or (out$ adj2 hospital$) or ambulat$ or same day or same-day).tw,kf. (335278)
10. (surgicenter$ or urgent care$).tw,kf. (2687)
11. Ambulatory Surgical Procedures/or (surg$ adj3 (day or (day$ adj3 case$) or short-stay$ or office-based)).tw,kf. (30079)
12. Elective Surgical Procedures/or ((elective$ or urgent) adj3 surg$).tw,kf. (41069)
13. (post-discharg$ or postdischarg$ or (discharg$ and (non-hospitali$ adj3 hospitali$))).tw,kf. (10712)
14. (((after$ or post$ or following) adj2 discharg$) and (non-hospitali$ or hospitali$)).tw,kf. (11629)
15. or/8-14 (466172)
16. 1 and 7 and 15 (180)

Database: Embase Classic + Embase <1947 to 2021 July 21>

Search Strategy:

1. (((exp Coronavirus/or exp Coronavirus Infections/or (coronavirus* or corona virus* or OC43 or NL63 or 229E or HKU1 or HCoV* or ncov* or covid* or sars-cov* or sarscov* or Sars-coronavirus* or Severe Acute Respiratory Syndrome Coronavirus* or D614G).mp.) not (SARS or SARS-CoV or MERS or MERS-CoV or Middle East respiratory syndrome or camel* or dromedar* or equine or coronary or coronal or covidence* or covidien or influenza virus or HIV or bovine or calves or TGEV or feline or porcine or BCoV or PED or PEDV or PDCoV or FIPV or FCoV or SADS-CoV or canine or CCov or zoonotic or avian influenza or H1N1 or H5N1 or H5N6 or IBV or murine corona*).mp.) or (((pneumonia or covid* or coronavirus* or corona virus* or ncov* or 2019-ncov or sars*).mp. or exp pneumonia/) and Wuhan.mp.) or (coronavirus disease 2019 or 2019-ncov or ncov19 or ncov-19 or 2019-novel CoV or severe acute respiratory syndrome coronavirus 2 or sars-cov2 or sars-cov-2 or sarscov2 or sarscov-2 or Sars-coronavirus2 or Sars-coronavirus-2 or SARS-like coronavirus* or coronavirus-19 or covid19 or covid-19 or covid 2019 or ((novel or new or nouveau) adj2 (CoV or nCoV or covid or coronavirus* or corona virus or Pandemi*2)) or ((covid or covid19 or covid-19) and pandemic*2) or (coronavirus* and pneumonia)).mp. or (coronavirus disease 2019 or severe acute respiratory syndrome coronavirus 2).sh,dj.) and 20191201:20301231.(dc). (166242)
2. exp venous thromboembolism/or exp vein thrombosis/or lung embolism/(245252)
3. (thrombos$ adj3 (venous or vein$1 or deep-vein$ or deep-venous)).tw,kw. (93484)
4. (thrombo?emboli$ adj3 (venous or vein$1)).tw,kw. (43635)

 © 2022. The Author(s).

5. ((pulmona$ or lung$) adj (embol$ or thrombo?embol$ or micro?embol$)).tw,kw. (75891)
6. (VTE or DVT or (thromb$ adj3 (generati$ or risk$1))).tw,kw. (89490)
7. or/2-6 (312062)
8. exp ambulatory care/or outpatient department/or health center/or outpatient/(310531)
9. (outpatient$ or out-patient$ or ((follow-up or followed or followup) adj3 clinic$1) or (out$ adj2 hospital$) or ambulat$ or same day or same-day).tw,kw. (550402)
10. (surgicenter$ or urgent care$).tw,kw. (4295)
11. ambulatory surgery/or (surg$ adj3 (day or (day$ adj3 case $) or short-stay$ or office-based)).tw,kw. (43232)
12. elective surgery/or ((elective$ or urgent) adj3 surg$).tw,kw. (67885)
13. (post-discharg$ or postdischarg$ or (discharg$ and (non-hospitali$ adj3 hospitali$))).tw,kw. (18540)
14. (((after$ or post$ or following) adj2 discharg$) and (non-hospitali$ or hospitali$)).tw,kw. (21744)
15. or/8-14 (778273)
16. 1 and 7 and 15 (363)

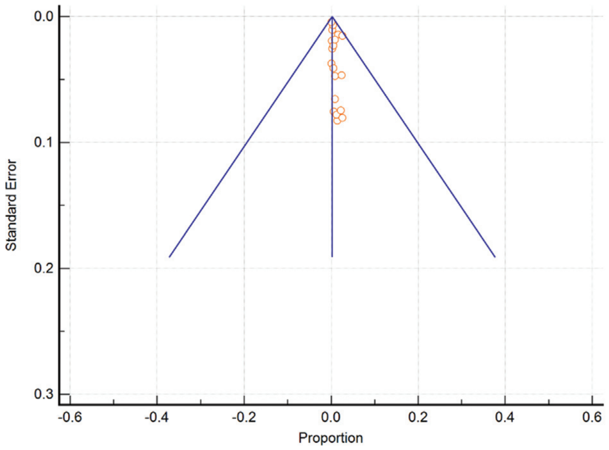

Fig. S1 Funnel plot for all patients (VTE events).

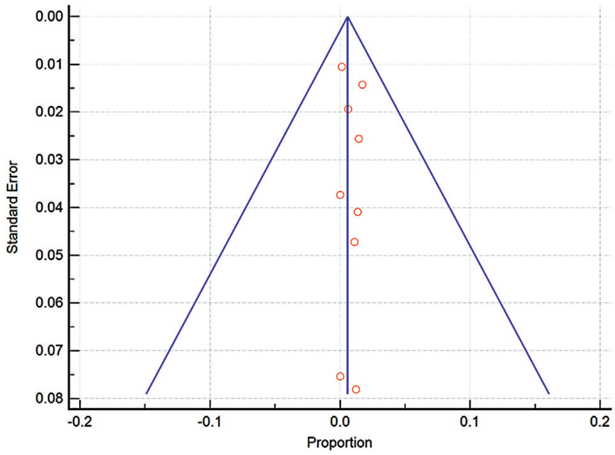

Fig. S2 Funnel plot for all patients (arterial events).

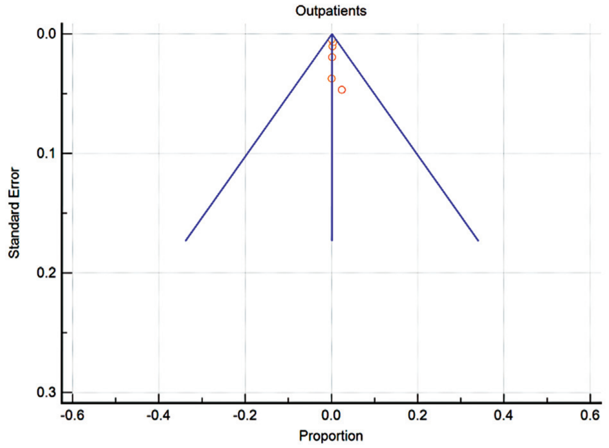

Fig. S3 Funnel plot for outpatients (VTE events).

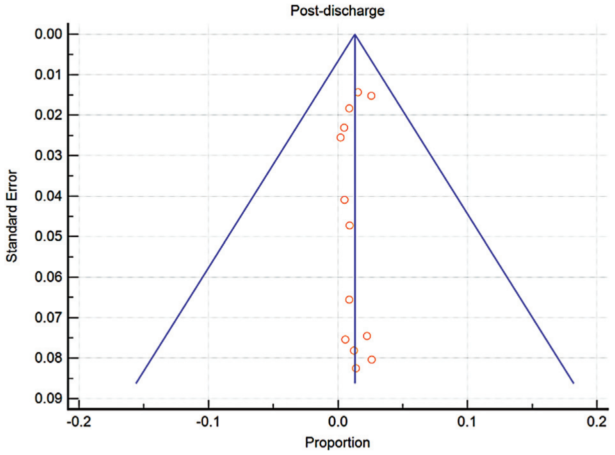

Fig. S4 Funnel plot for postdischarge patients (VTE events).

 © 2022. The Author(s).

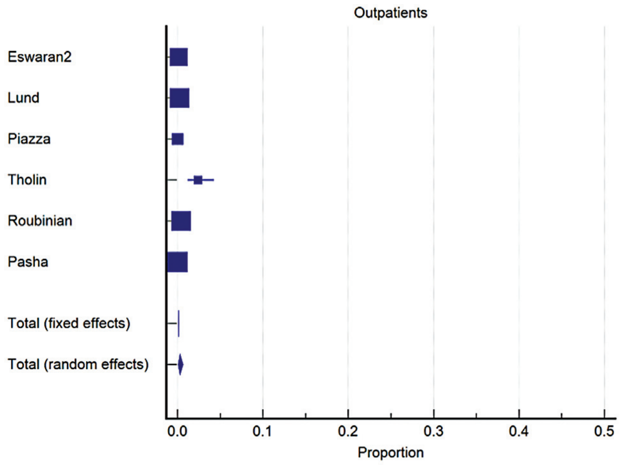

Fig. S5 Forest plot for outpatients (VTE events).

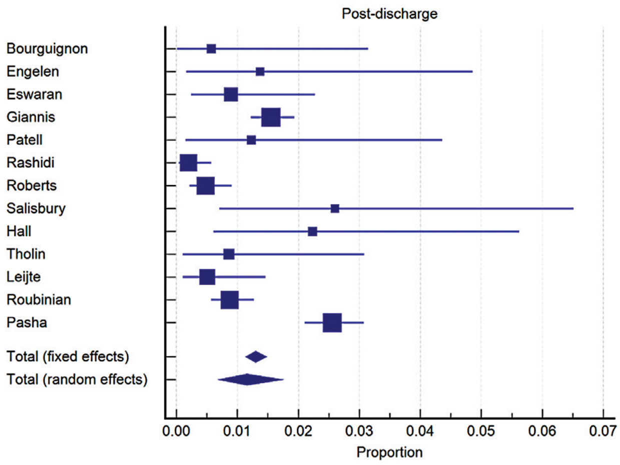

Fig. S6 Forest plot for post discharge patients (VTE events).

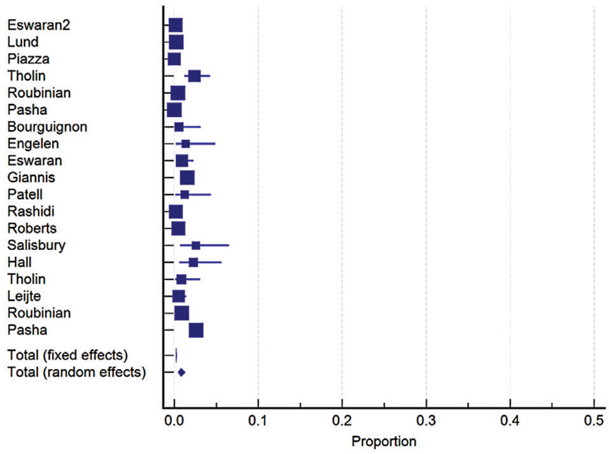

Fig. S7 Forest plot for all patients (VTE events).

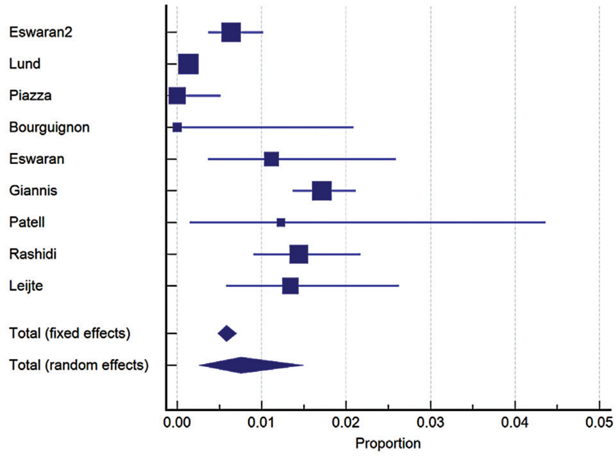

Fig. S8 Forest plot for all patients (arterial events).

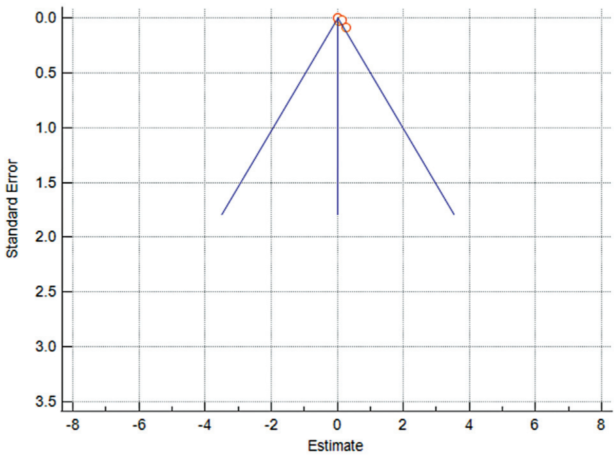

Fig. S9 Funnel plot for the incidence rate of venous thromboembolic events in outpatients.

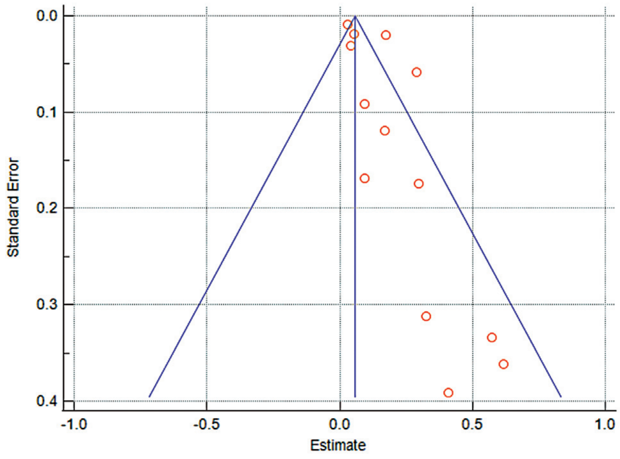

Fig. S10 Funnel plot for the incidence rate of venous thromboembolic events in post-discharge patients.

 © 2022. The Author(s).

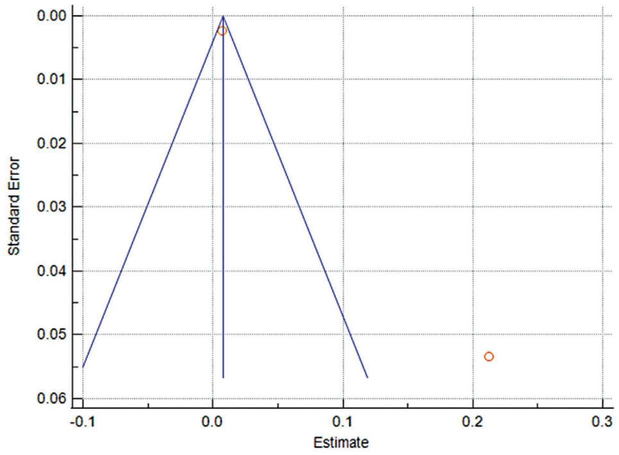

Fig. S11 Funnel plot for the incidence rate of arterial events in outpatients.

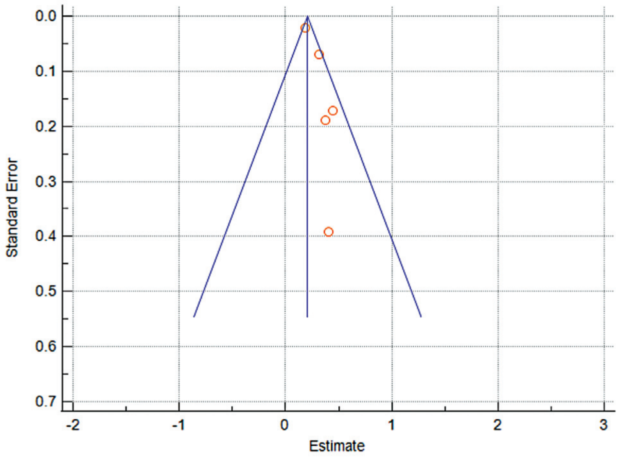

Fig. S12 Funnel plot for the incidence rate of arterial events in post-discharge patients.

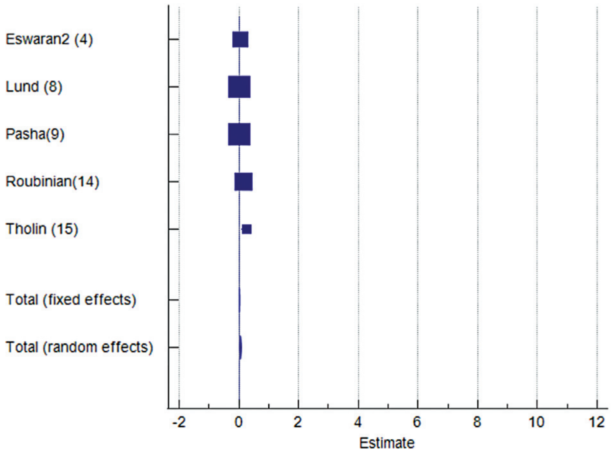

Fig. S13 Forest plot for the incidence rate of venous thromboembolic events in outpatients.

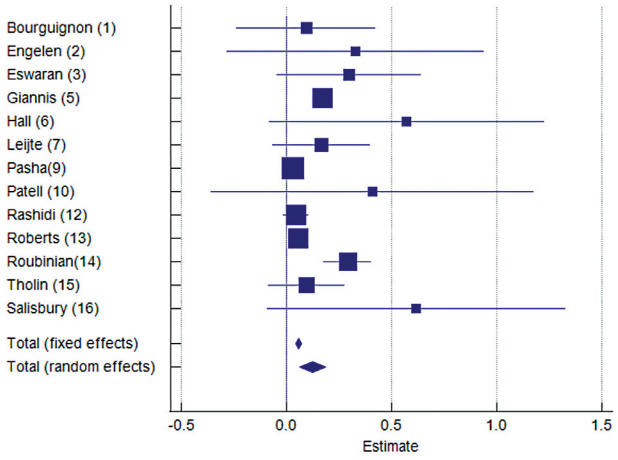

Fig. S14 Forest plot for the incidence rate of venous thromboembolic events in post-discharge patients.

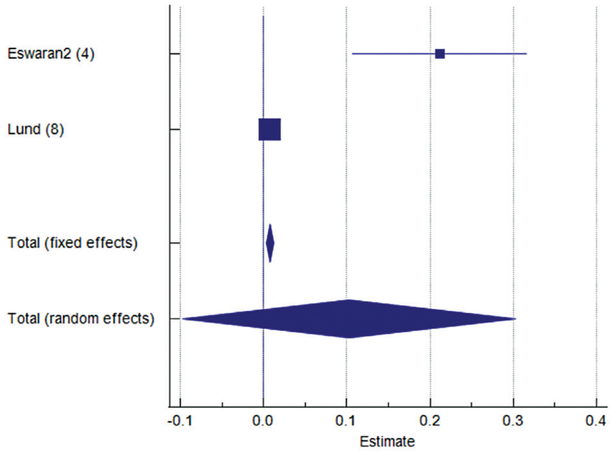

Fig. S15 Forest plot for the incidence rate of arterial events in outpatients.

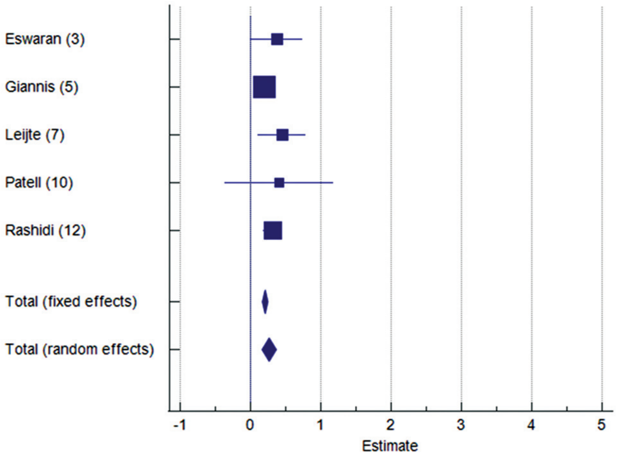

Fig. S16 Forest plot for the incidence rate of arterial events in post-discharge patients.

 © 2022. The Author(s).

Table S1 Characteristics of included studies

No.	Study	Study design	No. of discharged	No. of outpatients	Median age (y)	Thromboprophylaxis	VTE events	Arterial events	Median follow-up
1	Bourguignon et al[1]	Retrospective	175	–	63	No prophylaxis, 24 on AC already	1	0	60 days
2	Engelen et al[2]	Prospective	146	–	58	Low dose enoxaparin (0.5 mg/kg once daily) for 2 to 6 weeks after discharge was considered on individualized basis, especially in high-risk patients	2	–	6 weeks
3	Eswaran et al[3]	Retrospective	447	–	54	190 discharged on prophylaxis (DOAC in 90%)	4	5	30 days
4	Eswaran et al[4]	Retrospective	–	2,673	37.9	–	4	17	30 days
5	Giannis et al[5]	Prospective	4,906	–	61.7	Thromboprophylaxis was prescribed in 12.7% based on IMPROVE VTE score.	76	84	90 days
6	Hall et al[6]	Prospective	179	21	54.8	–	4	–	4-6 weeks
7	Leijte et al[7]	Retrospective	596			–	3	8	30 days
8	Lund et al[8]	Prospective	–	8,983	43	–	20	12	2 weeks to 6 months post positive COVID
9	Pasha et al[9]	Retrospective	4,340	50,013		–	11/111[a]	–	90 days before or after positive COVID
10	Patell et al[10]	Retrospective	163	–	58	No prophylaxis post discharge	2	2	30 days
11	Piazza et al[11]	Retrospective	–	715	44.28	1 patient on prophylaxis /16 patients on therapeutic AC	0	0	30 days
12	Rashidi et al[12]	Retrospective	1,529	–	56	97% on thromboprophylaxis	3	22	45 days
13	Roberts et al[13]	Retrospective	1,877	–	–	No outpatient thromboprophylaxis	9	–	90 days
14	Nareg et al[14]	Retrospective	2,984	22,209		–	26/95[a]	–	30 days
15	Tholin et al[15]	Prospective	232	458	49.6	19 patients discharged on thromboprophylaxis	2/11[a]	–	3 months
16	Salisbury et al[16]	Retrospective	154	–	61.5	5 patients got thromboprophylaxis, 51 already on full dose AC	4	–	42 days

Abbreviation: VTE, venous thromboembolism ; AC, Anticoagulants; DOAC, direct oral anticoagulants.
[a]Numbers represent events in post-discharged and outpatients, respectively.

 © 2022. The Author(s).

Table S2 Quality assessment of included studies according to the Newcastle-Ottawa scale

	Author	Study design	Selection	Compatibility	Outcome
1	Bourguignon et al[1]	Retrospective	***	*	***
2	Engelen et al[2]	Prospective	***	*	***
3	Eswaran et al[3]	Retrospective	****	*	***
4	Eswaran et al[4]	Retrospective	Abstract only		
5	Giannis et al[5]	Prospective	***	*	***
6	Hall et al[6]	Prospective	***	*	**
7	Leijte et al[7]	Retrospective	**	*	*
8	Lund et al[8]	Prospective	***	*	**
9	Pasha et al[9]	Retrospective	***	*	***
10	Patell et al[10]	Retrospective	***	*	***
11	Piazza et al[11]	Retrospective	***	*	***
12	Rashidi et al[12]	Retrospective	***	*	**
13	Roberts et al[13]	Retrospective	***	*	**
14	Nareg et al[14]	Retrospective	***	*	***
15	Tholin et al[15]	Prospective	***	*	***
16	Salisbury et al[16]	Retrospective	***	*	***

Table S3 Pooled estimates of the proportion of venous thromboembolic events according to proportion of thromboprophylaxis use and duration of follow-up

	Proportion	95% confidence interval	Q-test	*p*-Value	I^2
Thromboprophylaxis use					
> 20%	1.043	0.183–2.595	17.27	<0.001	82.63
< 20%	0.659	0.372–1.028	114.47	<0.001	91.26
Not reported	1.019	0.001–3.886	357.81	<0.001	99.16
Duration of follow-up					
> 42 days	0.809	0.230–1.737	560.9345	<0.001	98.57
< 42 days	0.665	0.373–1.041	42.3308	<0.001	78.74

 © 2022. The Author(s).

Table S4 Egger's tests for publication bias (*p*-value)

Outcome	Egger's tests (*p*-value)	No. of studies
Arterial events(discharged)	0.0793	6
Arterial event (outpatients)	0.8755	3
Arterial events (all patients)	0.5275	9
Arterial events (prospective studies)	<0.0001	2
Arterial events (retrospective studies)	0.9234	7
Arterial events IR (outpatients)	<0.001	2
Arterial events IR (discharged)	0.0499	5
VTE (discharged)	0.5498	13
VTE (outpatients)	0.3614	6
VTE (all patients)	0.0196	16
VTE (prospective studies)	0.3127	5
VTE (retrospective studies)	0.0857	11
VTE IR (outpatients)	0.2075	5
VTE IR (discharged)	0.1431	13

Abbreviations: IR, incidence rate; VTE, venous thromboembolism.

References

1 Bourguignon A, Beaulieu C, Belkaid W, Desilets A, Blais N. Incidence of thrombotic outcomes for patients hospitalized and discharged after COVID-19 infection. Thromb Res 2020; 196:491–493
2 Engelen MM, Vandenbriele C, Balthazar T, et al. Venous thromboembolism in patients discharged after COVID-19 hospitalization. Semin Thromb Hemost 2021;47(04):362–371
3 Eswaran H, Jarmul JA, Shaheen AW, et al. Vascular thromboembolic events following COVID-19 hospital discharge: Incidence and risk factors. Res Pract Thromb Haemost 2021;5(02): 292–295
4 Eswaran H, Jarmul J, Shaheen A, et al. Incidence of vascular thromboembolic events in outpatients with mild COVID-19. Blood 2020;136:31–32
5 Giannis D, Ziogas IA, Gianni P. Coagulation disorders in coronavirus infected patients: COVID-19, SARS-CoV-1, MERS-CoV and lessons from the past. J Clin Virol 2020;127:104362
6 Hall J, Myall K, Lam JL, et al. Identifying patients at risk of post-discharge complications related to COVID-19 infection. Thorax 2021;76(04):408–411
7 Leijte WT, Wagemaker NMM, van Kraaij TDA, et al. Mortality and re-admission after hospitalization with COVID-19 [in Dutch]. Ned Tijdschr Geneeskd 2020;164(49):D5423
8 Lund LC, Hallas J, Nielsen H, et al. Post-acute effects of SARS-CoV-2 infection in individuals not requiring hospital admission: a Danish population-based cohort study. Lancet Infect Dis 2021; 21(10):1373–1382
9 Pasha AK, McBane RD, Chaudhary R, et al. Timing of venous thromboembolism diagnosis in hospitalized and non-hospitalized patients with COVID-19. Thromb Res 2021;207:150–157
10 Patell R, Bogue T, Koshy A, et al. Postdischarge thrombosis and hemorrhage in patients with COVID-19. Blood 2020;136(11): 1342–1346
11 Piazza G, Campia U, Hurwitz S, et al. Registry of arterial and venous thromboembolic complications in patients with COVID-19. J Am Coll Cardiol 2020;76(18):2060–2072
12 Rashidi F, Barco S, Kamangar F, et al. Incidence of symptomatic venous thromboembolism following hospitalization for coronavirus disease 2019: Prospective results from a multi-center study. Thromb Res 2021;198:135–138
13 Roberts LN, Whyte MB, Georgiou L, et al. Postdischarge venous thromboembolism following hospital admission with COVID-19. Blood 2020;136(11):1347–1350
14 Nareg H, Roubinian M, Jennifer R, et al. Incidence of 30-day venous thromboembolism in adults tested for SARS-CoV-2 infection in an integrated health care system in Northern California. JAMA Intern Med 2021;181(07):997–999
15 Tholin B, Ghanima W, Einvik G, et al. Incidence of thrombotic complications in hospitalised and non-hospitalised patients after COVID-19 diagnosis. Br J Haematol 2021;194(03):542–546
16 Salisbury R, Iotchkova V, Jaafar S, et al. Incidence of symptomatic, image-confirmed venous thromboembolism following hospitalization for COVID-19 with 90-day follow-up. Blood Adv 2020;4 (24):6230–6239

 © 2022. The Author(s).
